# Evaluating biparametric MRI for diagnosing muscle-invasive bladder cancer with variant urothelial histology: a multicenter study

**DOI:** 10.1186/s40644-025-00831-x

**Published:** 2025-02-18

**Authors:** Peikun Liu, Lingkai Cai, Hongliang Que, Meihua Jiang, Xuping Jiang, Bo Liang, Gongcheng Wang, Linjing Jiang, Xiao Yang, Qiang Lu

**Affiliations:** 1 Department of Urology, The First Affiliated Hospital of Nanjing Medical University, Nanjing, 210029 China; 2 Department of Urology, Tongji Hospital, Tongji Medical College, Huazhong University of Science and Technology, Wuhan, 430030 China; 3 Department of Urology, The Affiliated Wuxi People’s Hospital of Nanjing Medical University, Wuxi People’s Hospital, Wuxi Medical Center, Nanjing Medical University, Wuxi, 214023 China; 4 Department of Urology, The Affiliated Suzhou Hospital of Nanjing Medical University, Suzhou Municipal Hospital, Suzhou, Jiangsu 215000 China; 5 Department of Radiology, Affiliated Hospital of Nanjing University of Traditional Chinese Medicine, Jiangsu Provincial Hospital of Traditional Chinese Medicine, Nanjing, 210029 China; 6 Department of Urology, Yixing People’s Hospital, Yixing, 214200 China; 7 Department of Radiology, Union Hospital, Tongji Medical College, Huazhong University of Science and Technology, Wuhan, 430022 China; 8 Department of Urology, The Affiliated Huai’an No. 1 People’s Hospital of Nanjing Medical University, Huai’an, Jiangsu 223300 China; 9 No. 300, Guangzhou Road, Gulou District, Nanjing City, Jiangsu Province China

**Keywords:** Variant histology, Vesical imaging reporting and data system, Biparametric MRI, Diagnostic performance

## Abstract

**Background:**

Vesical Imaging-Reporting and Data System (VI-RADS) based on multiparametric MRI (mp-MRI) demonstrated excellent performance in diagnosing muscle-invasive bladder cancer (MIBC) in cases of pure urothelial carcinoma. However, the performance of VI-RADS based on mp-MRI and biparametric MRI (bp-MRI) in diagnosing urothelial carcinoma with variant histology (VUC) remains unknown.

**Purpose:**

To evaluate the applicability of VI-RADS using mp-MRI and bp-MRI in diagnosing MIBC in patients with VUC.

**Methods:**

A retrospective analysis was conducted on 86 patients with VUC from different medical centers. Each patient underwent mp-MRI, with images evaluated using VI-RADS scores. The acquired images were divided into two groups: the mp-MRI group and the bp-MRI group. The mp-MRI group was evaluated according to the VI-RADS protocol. For the bp-MRI group, two VI-RADS scoring criteria were established: bp-DWI, primarily driven by DWI, and bp-T2WI, primarily driven by T2WI. The bp-MRI group was evaluated based on these two criteria. Inter-reader agreement performance was evaluated using Kappa analysis. The evaluation methods were evaluated by receiver operating characteristic curve. Comparison of the area under the curve (AUC) was performed used DeLong’s test. A p-value < 0.05 was considered significant.

**Results:**

Inter-reader agreement was high across all evaluation methods, with Kappa values exceeding 0.80. The AUCs for mp-MRI, bp-DWI, and bp-T2WI were 0.934, 0.885, and 0.932, respectively. The diagnostic performance of bp-T2WI was comparable with that of mp-MRI (*p* = 0.682) and significantly higher than bp-DWI (*p* = 0.007). Both mp-MRI and bp-T2WI demonstrated high sensitivity and specificity.

**Conclusion:**

VI-RADS based on mp-MRI demonstrates good diagnostic performance for MIBC in VUC patients. bp-T2WI may provide comparable diagnostic performance to mp-MRI.

**Supplementary Information:**

The online version contains supplementary material available at 10.1186/s40644-025-00831-x.

## Introduction

Bladder cancer is one of the most common types of cancer, mainly urothelial carcinoma [1]. Urothelial carcinoma with variant histology (VUC), including urothelial carcinoma with divergent differentiation, micropapillary, microcystic, nested patterns, and other variants, comprises for 10-25% of all bladder cancer cases [2–4]. Compared to pure urothelial carcinoma, VUCs are reported to have a greater tendency to present in muscle-invasive bladder cancer (MIBC) and exhibit poorer responses to radiotherapy and chemotherapy [5–9].

Currently, the gold standard for diagnosing MIBC is transurethral resection of bladder tumor (TURBT). However, TURBT has limitations, such as being invasive and carrying the risk of tumor understaging. A recent meta-analysis reveals that 4.5-4.8% of tumors initially classified as Ta or T1 stage during the initial TURBT may progress to MIBC upon repeat TURBT [10]. Therefore, a primary focus in the diagnosis and treatment of bladder cancer is the development of a non-invasive and precise staging tool.

In 2018, Vesical Imaging-Reporting and Data System (VI-RADS) based on multiparametric MRI (mp-MRI) was introduced, offering a standardized, non-invasive approach for imaging and reporting in bladder cancer [11]. Many studies have reported its excellent inter-reader consistency and diagnostic performance [12, 13]. One study has reported that VI-RADS is equally effective for VUC compared to pure urothelial carcinoma [14]. However, due to the lack of supporting evidence from other studies, additional validation is necessary to confirm this conclusion. Moreover, VI-RADS extends scan time due to its inclusion of dynamic contrast-enhanced (DCE) imaging and carries associated risks related to the intravenous administration of gadolinium-based contrast agents [15–17]. Recent Studies have indicated that biparametric MRI (bp-MRI) may provide comparable diagnostic performance to mp-MRI in detecting MIBC [18]. However, uncertainty persists regarding the effectiveness of bp-MRI for VUC. While one study has recommended mp-MRI-based methods over bp-MRI for evaluating muscle invasiveness of VUC, further evaluation and establishment of a more comprehensive criterion for bp-MRI are warranted [19].

The objective of this study is to evaluate the applicability of VI-RADS based on both mp-MRI and bp-MRI in VUC. In addition, we established two sets of criteria for bp-MRI evaluation in the absence of DCE: one primarily based on T2-weighted imaging (T2WI) and the other on diffusion-weighted imaging (DWI). Our goal is to determine which criterion is more suitable for evaluating VUC.

**Fig. 1:**
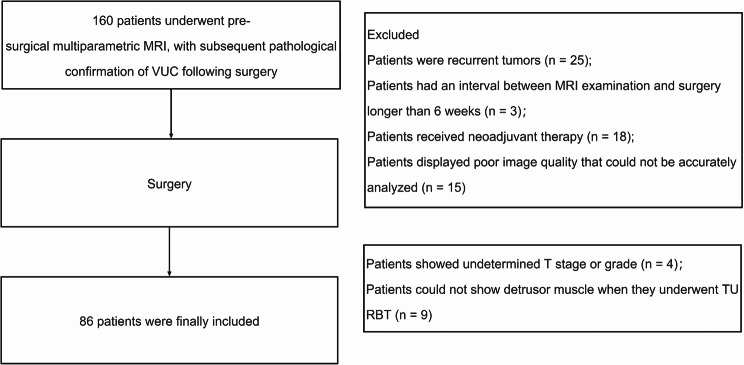
Study flowchart

## Materials and methods

Given the retrospective design of this study, the requirement for ethical approval was waived by the institutional review board. Written informed consent was obtained from patients for the use of their relevant information.

### Patient population

A retrospective analysis was performed on 160 patients whose pathological specimens revealed VUC following surgery at six medical centers. Before undergoing surgery, all patients had undergone mp-MRI examination. Histologic grade was evaluated according to the 2004 World Health Organization of urological pathology classification. T stage was evaluated according to the 2017 Union for International Cancer Control of TNM classification.

The following patients were excluded: (a) 25 patients were recurrent tumors; (b) 3 patients had an interval between mp-MRI examination and surgery longer than 6 weeks; (c) 18 patients received neoadjuvant therapy; (d) 15 patients displayed poor image quality that could not be accurately analyzed for reasons such as severe artifacts, inadequate bladder filling, and an inability to identify tumors; (e) 4 patients showed undetermined T stage or grade in postoperative pathology; (f) 9 patients could not show detrusor muscle when they underwent TURBT. The final study group consisted of a total of 86 patients. The flowchart illustrating the selection process for the study population is depicted in Fig. 1.

**Fig. 2:**
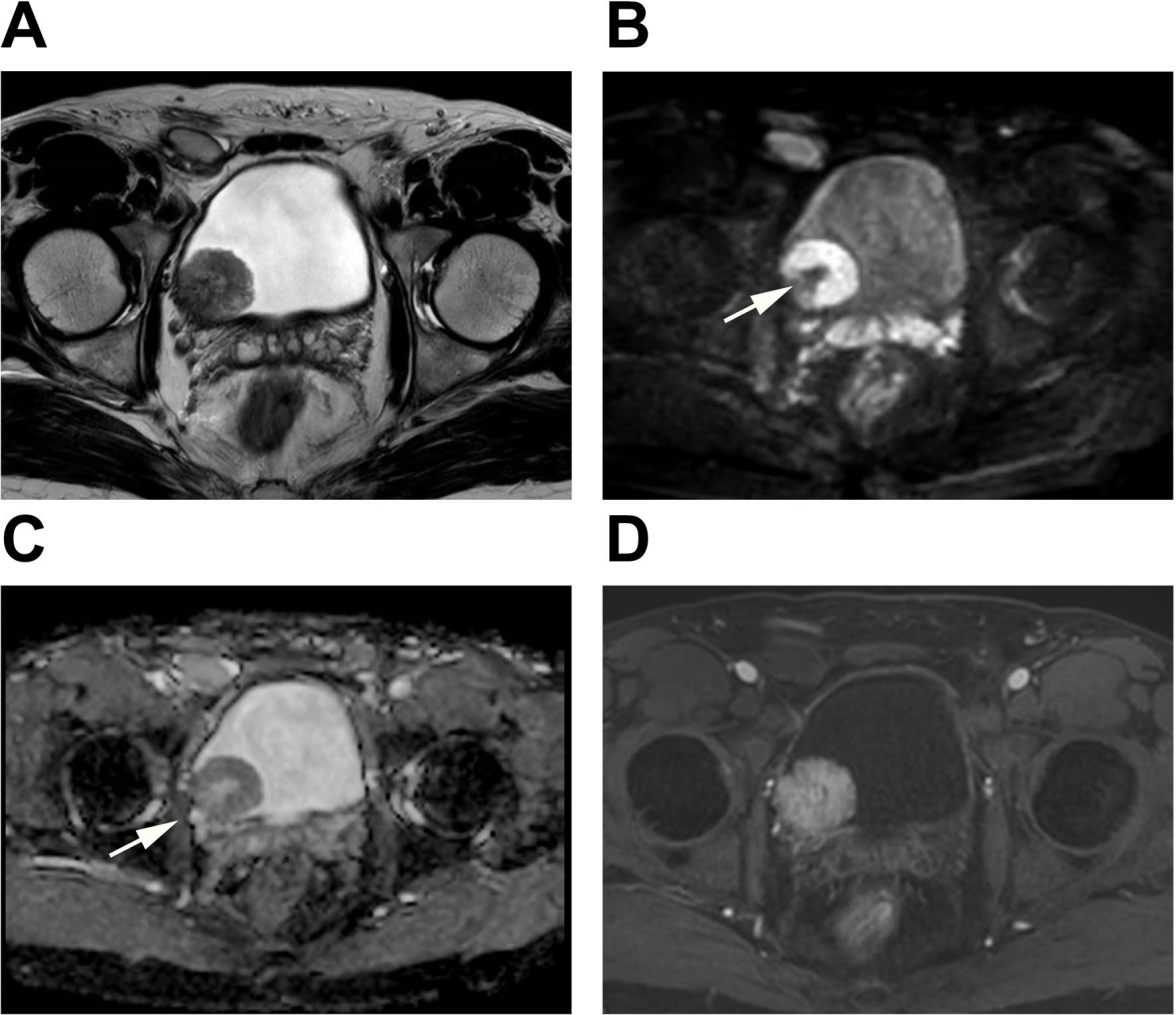
A case was evaluated using three evaluation methods. A patient over 40 years old underwent mp-MRI before primary TURBT. T2WI revealed a single tumor with a clearly defined high signal intensity stalk on the right posterior wall of the bladder. Tumors with a clear stalk were also identified on DWI and the ADC map (Arrow). All readers assigned a T2WI score of 2 and a DWI score of 2, resulting in both bp-DWI and bp-T2WI VI-RADS scores being 2. DCE did not show early enhancement of the muscularis propria. Therefore, all the readers assigned a DCE score of 2, resulting in a VI-RADS score of 2 for mp-MRI. T stage after TURBT was T1-high grade with squamous differentiation. **A**: T2WI; **B**: DWI; **C**: ADC; **D**: DCE

**Fig. 3:**
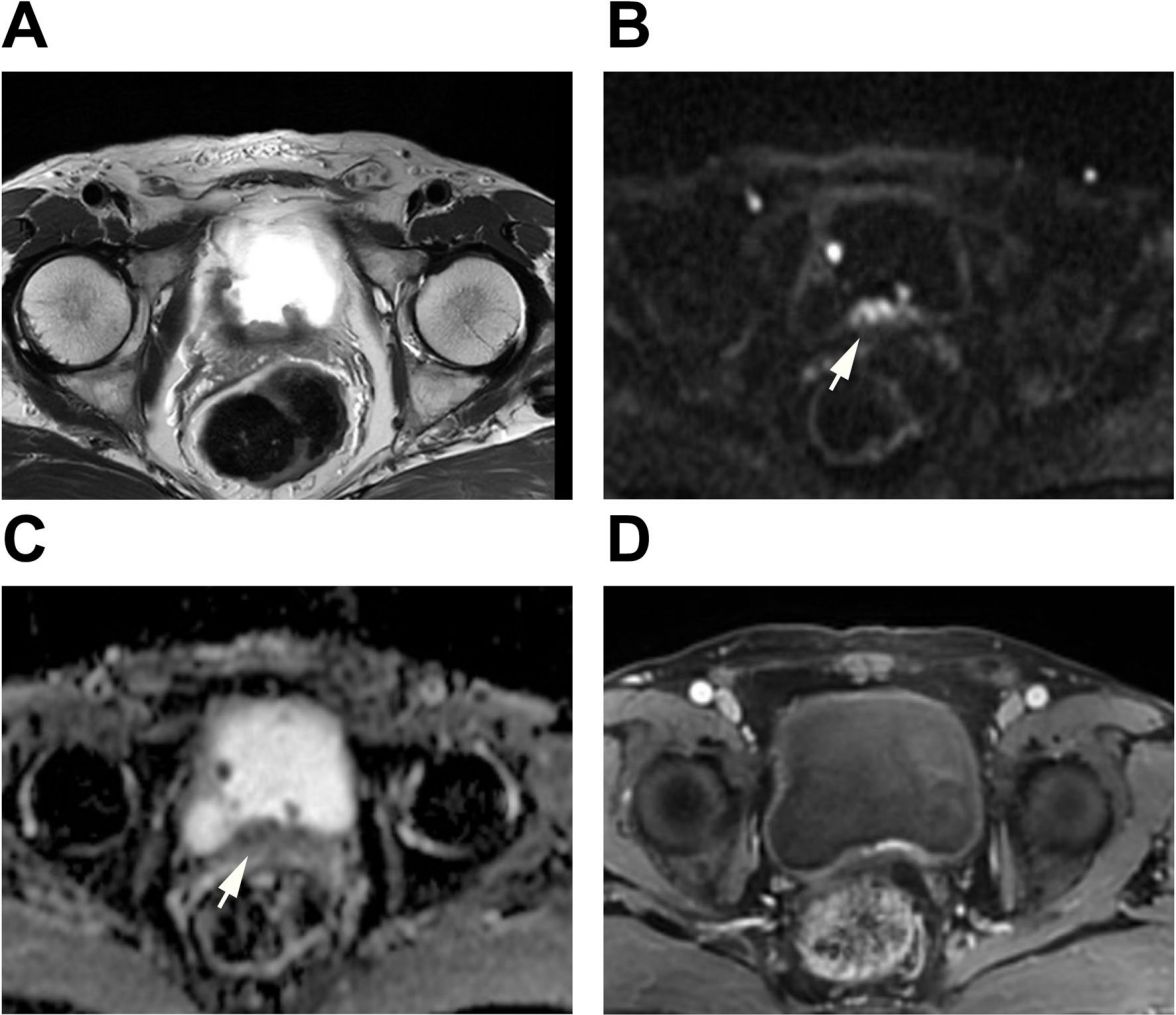
A case was evaluated using three evaluation methods. A patient over 65 years old with hematuria underwent mp-MRI prior to the initial TURBT. T2WI showed multiple sessile tumors on the trigone, dome, right wall, and posterior wall of bladder. The tumor located on the trigone, possessing the highest VI-RADS score, was selected for analysis. Clear stalks and thickened inner layers were observed on DWI and ADC map (Arrow). All readers assigned a T2WI score of 3 and a DWI score of 2, resulting in bp-DWI VI-RADS score was 2 and bp-T2WI VI-RADS score was 3. DCE did not show any clear disruption of muscularis propria. Therefore, all the readers assigned a DCE score of 3, resulting in a VI-RADS score of 3 for mp-MRI. T stage after TURBT was T2-high grade with glandular differentiation. The pathology results from the subsequent radical cystectomy confirmed this observation. **A**: T2WI; **B**: DWI; **C**: ADC; **D**: DCE

**Fig. 4:**
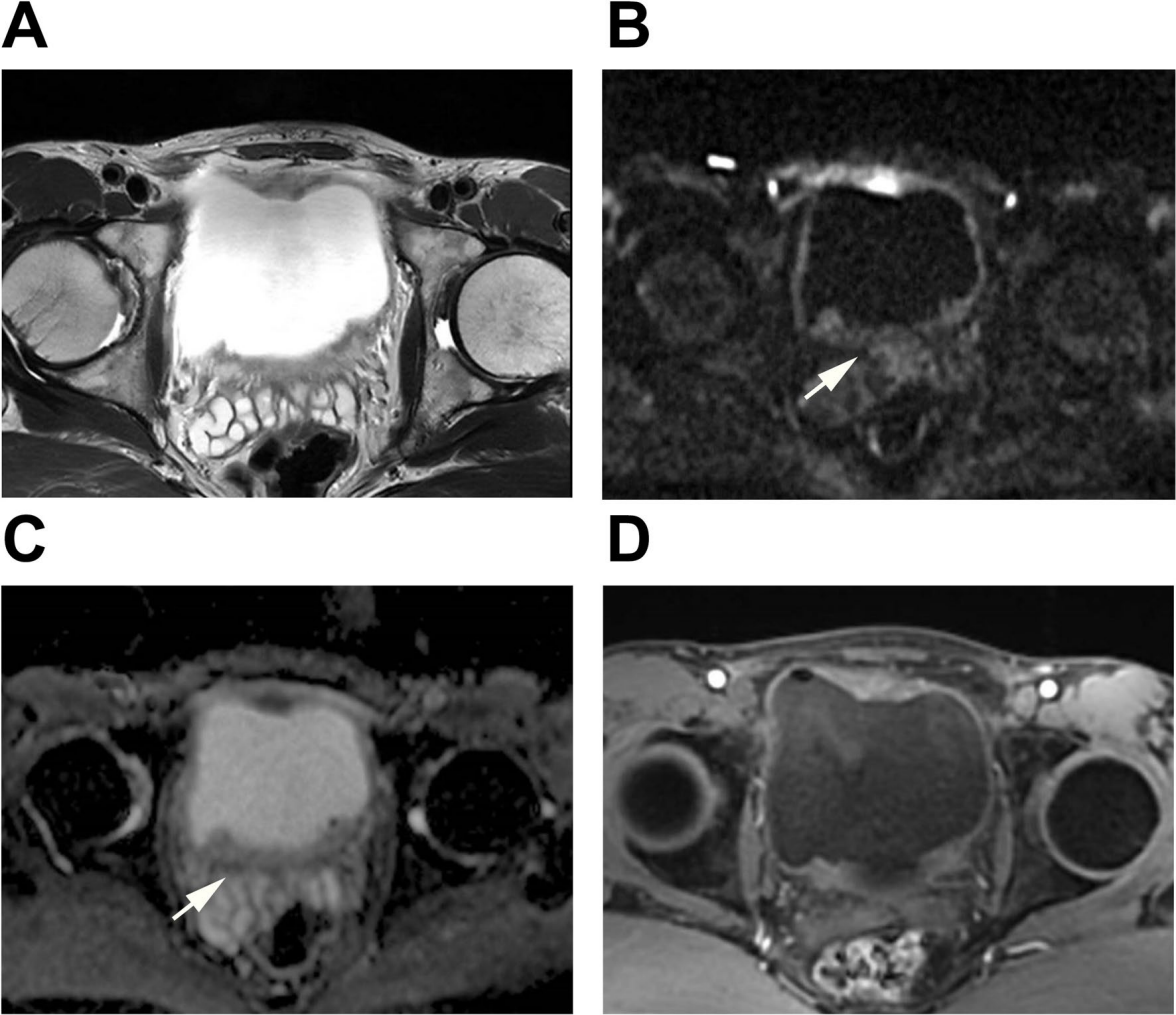
A case was evaluated using three evaluation methods. A patient over 65 years old with hematuria underwent mp-MRI prior to radical cystectomy. T2WI showed a sessile tumor on the anterior wall of bladder with interruption of low signal intensity. The presence of a tumor extending focally into the muscularis propria was observed on DWI and ADC map (Arrow). All readers assigned a T2WI score of 4 and a DWI score of 4, resulting in both bp-DWI and bp-T2WI VI-RADS scores being 4. DCE showed early enhancement of the tumor extending focally to muscularis propria. Therefore, all the readers assigned a DCE score of 4, resulting in a VI-RADS score of 4 for mp-MRI. T stage after radical cystectomy was T4-high grade with squamous differentiation. **A**: T2WI; **B**: DWI; **C**: ADC; **D**: DCE

**Fig. 5:**
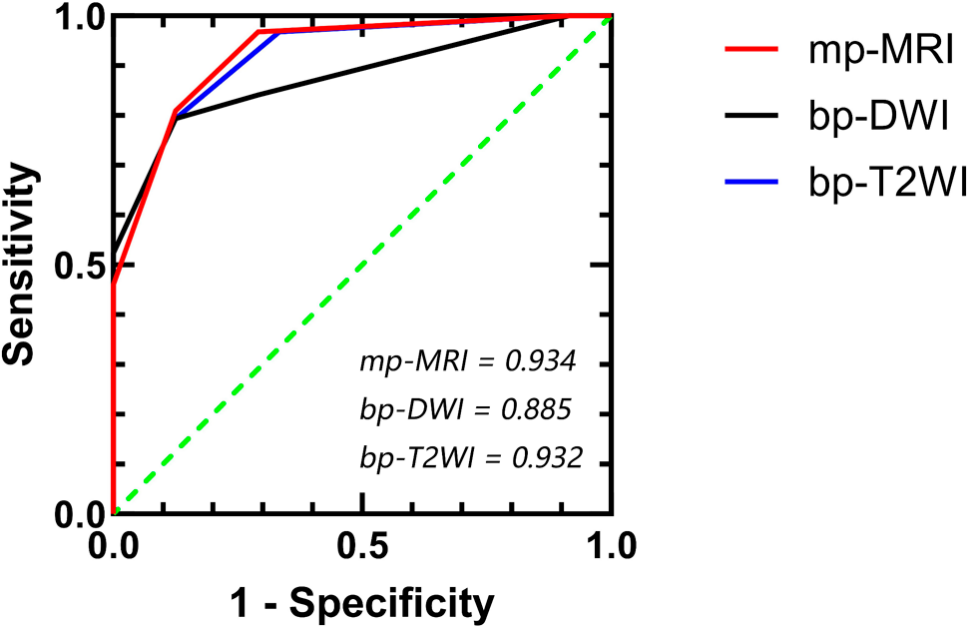
Receiver operating characteristic curves of the three evaluation methods

### Patient characteristics

Among 86 patients diagnosed with VUC, 70 were male (81.4%) and 16 were female (18.6%), with a median age of 65 years (interquartile range: 60–75 years). Regarding surgical treatment, 19 patients (22.1%) underwent TURBT, 64 patients (74.4%) underwent radical cystectomy, and 3 patients (3.5%) underwent partial cystectomy. Specifically, there were 4 cases in Ta stage (4.7%), 20 cases in T1 stage (23.3%), 25 cases in T2 stage (29.1%), 30 cases in T3 stage (34.9%), and 7 cases in T4 stage (8.1%). In addition, 3 patients (3.5%) had low-grade tumors, and 83 patients (96.5%) had high-grade tumors. The clinical details of VUC patients are presented in Table 1.

**Table 1:** The clinical details of VUC patients

Parameter	*N* (%)
Gender	
Male	70 (81.4)
Female	16 (18.6)
Age	65 (IQR: 60–75)
Operation	
TURBT	19 (22.1)
Radical cystectomy	64 (74.4)
Partial cystectomy	3 (3.5)
T stage	
Ta	4 (4.7)
T1	20 (23.3)
T2	25 (29.1)
T3	30 (34.9)
T4	7 (8.1)
Histological grade	
Low grade	3 (3.5)
High grade	83 (96.5)
Classification of VUC	
Squamous differentiation	44 (51.2)
Glandular differentiation	15 (17.4)
Micropapillary	7 (8.1)
Plasmacytoid	3 (3.5)
Sarcomatoid	6 (7.0)
Nested	1 (1.2)
Small cell carcinoma	5 (5.8)
Squamous differentiation + glandular differentiation	3 (3.5)
Squamous differentiation + plasmacytoid	1 (1.2)
Squamous differentiation + glandular differentiation + micropapillary	1 (1.2)

### MRI protocol

The MRI protocol used at our center is outlined below, while the MRI protocols from other centers are presented in Supplementary Table 1. MRI scans were performed in a 3.0 T MRI system (UIH uMR 770; Shanghai, China) with the use of a 48-channel phased-array surface coil. Transverse T2WI were performed with a turbo spin-echo sequence [repetition time/echo time (TR/TE): 3400/100 ms, acquisition matrix 288\0\0\288, in-plane spatial resolution: 0.5 × 0.5 mm, slice thickness: 4.0 mm, slice gap: 0.8 mm, and field of view (FOV): 216 mm]. DWI was conducted with an axial breathing-free spin-echo EPI sequence (TR/TE 6500/83 ms, flip angle 90, acquisition matrix 128\0\0\99, slice thickness: 5.0 mm, and FOV: 300 mm). *b* value was set between 0 and 2000 s/mm2 (0, 50, 700, 1400, and 2000). DCE was performed with a sequence (TR/TE 3.1/1.4 ms, flip angle 10, acquisition matrix 176\0\0\120, in-plane spatial resolution 1.06 × 1.06 mm, slice thickness 2.6 mm, FOV 240 mm, and acquisition duration 2116 s). A single body weight-based dose (0.1 mmol/kg) of gadolinium-based contrast agent (Magnevist, Bayer Healthcare) was intravenously injected at a constant rate of 2.5 mL/second after the fifth dynamic scan, followed by a flush of 25 mL of saline at a flow rate of 2.5 mL/second.

**Table 2:** Evaluation methods for VI-RADS based on biparametric MRI

T2WI score	DWI score	VI-RADS based on biparametric MRI	
		bp-DWI	bp-T2WI
1	1	1	1
2	2	2	2
3	2	2	3
3	3	3	3
3	4	4	4
4	3	4	4
4	4	4	4
5	3	4	4
5	4	4	4
5	5	5	5

T2WI: T2-weighted images; DWI: diffusion-weighted imaging; DCE: dynamic contrast-enhanced; VI-RADS: Vesical Imaging-Reporting and Data System

### VI-RADS evaluation

All images were analyzed using RadiAnt Dicom Viewer (Version 2021.1, Medixant, Poznan, Poland). The collected images were divided into two groups: the bp-MRI group, which included transverse, sagittal, and coronal T2WI along with transverse DWI; and the mp-MRI group, which additionally included transverse DCE. Images from both groups were independently evaluated by three readers with varying levels of experience: Reader 1 with over 3 years of experience, Reader 2 with over 5 years of experience, and Reader 3 with over 10 years of experience. The evaluations were conducted in two separate reading sessions, spaced at least 4 weeks apart to avoid recall bias. All readers were blinded to the pathology results. For patients with multiple tumors, the tumor with the highest VI-RADS score was selected for evaluation. In cases of discrepancies in VI-RADS score readings among the three initial readers, the final score was determined by Reader 4, who has over 30 years of experience.

The mp-MRI group was evaluated according to the VI-RADS protocol [11]. For the bp-MRI group, two scoring criteria based on the VI-RADS were established: bp-DWI, primarily driven by DWI, where the final VI-RADS score was determined by the DWI score when there was a discrepancy between the T2WI and DWI scores; and bp-T2WI, primarily driven by T2WI, where the final VI-RADS score was determined by the T2WI score when there was a discrepancy between the T2WI and DWI scores. The evaluation of the bp-MRI group was carried out using these two criteria. Detailed descriptions of these two bp-MRI criteria are provided in Table 2. Additionally, we also analyzed the performance of T2WI alone and DWI alone. Examples of reader evaluations are illustrated in Figs. 2 and 3, and Fig. 4.

### Statistical analysis

Statistical analyses were performed in IBM SPSS software, version 26 (IBM Corporation, Armonk, New York, USA). VI-RADS score ≥ 3 and ≥ 4 served as the cutoff values for diagnose MIBC, respectively. For the evaluation of VI-RADS score, the inter-reader agreement performance was evaluated using Cohen’s Kappa analysis. Area under the curve (AUC), sensitivity, specificity, positive predictive value (PPV), and negative predictive value (NPV) were used to evaluate each criterion. The AUCs of receiver operating characteristic (ROC) curve comparison was performed using the DeLong test in MedCalc software, version 20 (MedCalc Software Ltd, Acacialaan, Ostend, Belgium). *p* < 0.05 was considered significant.

## Results

The three evaluation methods include mp-MRI, bp-DWI, and bp-T2WI. Cohen’s Kappa values for mp-MRI, bp-DWI, and bp-T2WI were 0.861, 0.813, and 0.903, respectively, using a cutoff value of VI-RADS ≥ 3, and 0.881, 0.881, and 0.904, respectively, using a cutoff value of VI-RADS ≥ 4.

For patients evaluated by mp-MRI, VI-RADS scores were distributed as follows: 2 cases (2.3%) with a score of 1, 16 cases (18.6%) with a score of 2, 14 cases (16.3%) with a score of 3, 25 cases (29.1%) with a score of 4, and 29 cases (33.7%) with a score of 5. For patients evaluated by bp-DWI, the distribution was 2 cases (2.3%) with a score of 1, 24 cases (27.9%) with a score of 2, 7 cases (8.1%) with a score of 3, 20 cases (23.3%) with a score of 4, and 33 cases (38.4%) with a score of 5. For bp-T2WI, the scores were 2 cases (2.3%) with a score of 1, 15 cases (17.4%) with a score of 2, 16 cases (18.6%) with a score of 3, 20 cases (23.3%) with a score of 4, and 33 cases (38.4%) with a score of 5. The VI-RADS scores for the evaluation methods in diagnosing MIBC are shown in Table 3.

**Table 3:** VI-RADS score for evaluation methods in diagnosing muscle-invasive bladder cancer

VI-RADS Score	mp-MRI		bp-DWI		bp-T2WI	
	NMIBC	MIBC	NMIBC	MIBC	NMIBC	MIBC
VI-RADS 1	2	0	2	0	2	0
VI-RADS 2	15	1	15	9	14	1
VI-RADS 3	4	10	4	3	5	11
VI-RADS 4	3	22	3	17	3	17
VI-RADS 5	0	29	0	33	0	33

**Table 4:** Diagnostic performance of the three evaluation methods at different cutoff values

Methods	Sensitivity (95% CI)		Specificity (95% CI)		PPV (95% CI)		NPV (95% CI)		AUC	Comparison of ROC curves	*P* value
	≥ 3	≥ 4	≥ 3	≥ 4	≥ 3	≥ 4	≥ 3	≥ 4		mp-MRI vs. T2WI	0.682
mp-MRI	98.4 (91.3–100.0)	82.3 (70.5–90.8)	70.8 (48.9–87.4)	87.5 (67.6–97.3)	89.7 (82.4–94.2)	94.4 (85.4–98.0)	94.4 (70.5–99.2)	65.6 (52.2–76.9)	0.934	mp-MRI vs. DWI	0.171
										mp-MRI vs. bp-DWI	**0.008**
T2WI	98.4 (91.3–100.0)	80.7 (68.6–89.6)	66.7 (44.7–84.4)	87.5 (67.6–97.3)	88.4 (81.2–93.1)	94.3 (85.2–98.0)	94.1 (69.2–99.1)	63.6 (50.7–74.8)	0.932	mp-MRI vs. bp- T2WI	0.682
										T2WI vs. DWI	0.171
DWI	85.5 (74.2–93.1)	77.4 (65.0-87.1)	70.8 (48.9–87.4)	100.0 (85.8–100.0)	88.3 (80.1–93.4)	100.0	65.4 (49.5–78.5)	63.2 (52.0–73.1)	0.901	T2WI vs. bp-DWI	**0.007**
										T2WI vs. bp-T2WI	1.000
bp-DWI	85.5 (74.2–93.1)	80.7 (68.6–89.6)	70.8 (48.9–87.4)	87.5 (67.6–97.3)	88.3 (80.1–93.4)	94.3 (85.2–98.0)	65.4 (49.5–78.5)	63.6 (50.7–74.8)	0.885	DWI vs. bp-DWI	0.166
										DWI vs. bp-T2WI	0.171
bp-T2WI	98.4 (91.3–100.0)	80.7 (68.6–89.6)	66.7 (44.7–84.4)	87.5 (67.6–97.3)	88.4 (81.2–93.1)	94.3 (85.2–98.0)	94.1 (69.2–99.1)	63.6 (50.7–74.8)	0.932	bp-DWI vs. bp-T2WI	**0.007**

mp-MRI: multiparametric magnetic resonance imaging; PPV: positive predictive value; NPV: negative predictive value; AUC: area under curve

The diagnostic performance of the five evaluation methods for diagnosing MIBC is shown in Table 4. The AUCs for mp-MRI, T2WI, DWI, bp-DWI and bp-T2WI were 0.934, 0.932, 0.901, 0.885 and 0.932, respectively. There was no significant difference between mp-MRI and bp-T2WI (AUC: 0.934 vs. 0.932, *p* = 0.682). However, the AUCs for mp-MRI and bp-T2WI were significantly higher than that of bp-DWI (mp-MRI vs. bp-DWI, AUC: 0.934 vs. 0.885, *p* = 0.008; bp-T2WI vs. bp-DWI, AUC: 0.932 vs. 0.885, *p* = 0.007). The ROC curve analyses for mp-MRI, bp-DWI and bp-T2WI are illustrated in Fig. 5.

## Discussion

Previous studies have indicated that VUC is associated with advanced tumor stage, extravesical disease, and lymph node invasion [20–24]. In addition, research has reported that VUCs typically present as large and diffuse tumors, often exhibiting high VI-RADS scores on MRI [25]. In our study, we found that the proportion of MIBC in VUC cases was 70.9%, significantly higher than that observed in the overall bladder cancer population. Among these MIBC VUC cases, 35.4% were classified as T3 and 8.9% as T4 stages, indicating a notably higher degree of invasiveness in VUC. These findings are consistent with previous research, underscoring the aggressive nature of VUC. Therefore, accurate non-invasive staging of VUC is crucial for selecting appropriate treatment strategies.

Arita et al. [14] conducted a retrospective study on the diagnostic performance of VI-RADS in VUC. Compared with pure urothelial carcinoma, their study demonstrated that the AUC for VUC ranged from 0.89 to 0.92, with no significant difference between the PUC and VUC groups. This study demonstrated that the AUC of VI-RADS in diagnosing MIBC in VUC was 0.932. Although not directly compared with pure urothelial carcinoma, it also indicated good diagnostic performance of VI-RADS.

Compared to mp-MRI, bp-MRI has garnered significant attention as a more convenient and cost-effective examination method. Some studies have investigated the feasibility of using bp-MRI instead of mp-MRI for preoperative diagnostic evaluation of MIBC [26–29]. These research results suggest that bp-MRI can maintain a high diagnostic performance comparable to mp-MRI. However, it remains unknown whether this conclusion is equally applicable in VUC. In addition, when T2WI scores 3 and DWI scores 2, the VI-RADS score becomes uncertain [11]. Previous studies have generally relied on the referenced “bp-DWI” for evaluating VI-RADS, without specifically exploring whether the final VI-RADS scores should be categorized as 2 or 3 in this particular context [26–29]. Arita et al. [19] evaluated the diagnostic performance of bp-MRI for diagnosing MIBC in VUC and found that bp-MRI based on bp-DWI was significantly less effective than mp-MRI. In contrast, we simultaneously evaluated “bp-DWI”-based bp-MRI and “bp-T2WI”-based bp-MRI in assessing VUC. Our results indicated that while bp-DWI is inferior to mp-MRI, bp-T2WI was comparable to mp-MRI in evaluating VUC.

In this study, the AUC for bp-DWI was significantly lower than that of mp-MRI, whereas the AUC for bp-T2WI did not differ significantly from mp-MRI. One possible reason is that T2WI offers superior spatial resolution for anatomical morphology compared to DWI [11]. Furthermore, since VUC has a higher likelihood of muscle invasion compared to pure urothelial carcinoma, the enhanced spatial resolution of T2WI may improve the detection of tumors with muscle invasion, extension beyond the extravesical fat, or involvement of adjacent organs. In addition, T2WI significantly increases the sensitivity for diagnosing MIBC while only slightly decreasing its specificity compared to DWI, thereby improving its overall diagnostic performance. This improvement may be attributed to the lower contrast difference between tumor and muscle layers observed on DWI compared to T2WI [30].

Moreover, plasmacytoid urothelial carcinoma, a subtype of VUC, is characterized by diffuse and sizable tumor masses and is often associated with high VI-RADS scores on MRI due to its unique pathological and imaging features [31–34]. Consistent with previous studies, this study found that three out of four plasmacytoid-associated VUC cases were MIBC, and both mp-MRI and bp-MRI demonstrated good diagnostic performance in identifying these specific features and tumor invasion.

However, it should be noted that there are a few key differences between our study and that of Arita et al. First, the higher proportion of MIBC among VUC patients in our study may enhance T2WI performance, given its inherent tendency to overestimate VI-RADS scores. Second, their DWI data included both axial and sagittal images, thereby potentially providing more comprehensive information. Finally, to ensure pathological accuracy, high-risk patients in their study typically undergo a second TURBT within 4–6 weeks after the initial procedure. In contrast, we excluded patients whose initial TURBT lacked detrusor muscle information rather than performing a second TURBT, which may introduce potential selection bias.

This study has a few limitations. First, while the results support the use of bp-T2WI for diagnosing MIBC in VUC, its efficacy in pure urothelial carcinoma remains uncertain, and whether it maintains diagnostic performance comparable to mp-MRI is yet to be determined. Second, DWI and DCE in this study only included transverse images, which may slightly limit the comprehensiveness of the VI-RADS evaluation. In addition, the evaluation results may be influenced by the experience of readers. Finally, it is important to acknowledge that this study is a retrospective analysis with limited evidence. In particular, the low incidence of VUC has resulted in a small sample size, which may limit the generalizability and robustness of the findings. Therefore, future multi-center, large-scale prospective studies are necessary to further validate the conclusions of this study.

## Conclusions

VI-RADS based on mp-MRI demonstrates good diagnostic performance for MIBC in VUC patients. Replacing mp-MRI with bp-MRI based on the bp-T2WI criterion, which is primarily driven by T2WI, may result in diagnostic performance comparable to that of mp-MRI.

## Electronic supplementary material

Below is the link to the electronic supplementary material.

Supplementary Material 1

## Data Availability

No datasets were generated or analysed during the current study.

## Abbreviations

VUC: urothelial carcinoma with variant histology
NMIBC: non-muscle-invasive bladder cancer
MIBC: muscle-invasive bladder cancer
TURBT: transurethral resection of bladder tumor
VI-RADS: Vesical Imaging-Reporting and Data System
mp-MRI: multiparametric magnetic resonance imaging
bp-MRI: biparametric magnetic resonance imaging
DCE: dynamic contrast-enhanced
T2WI: T2-weighted images
DWI: diffusion-weighted imaging
ROC: receiver operating characteristic
AUC: area under curve
