# Supplementary material for: Evaluating biparametric MRI for diagnosing muscle-invasive bladder cancer with variant urothelial histology: a multicenter study
Source: Cancer Imaging. 2025 Feb 18;25:15. doi: 10.1186/s40644-025-00831-x (PMC11834218; doi:10.1186/s40644-025-00831-x)
Supplement: Supplementary file 1 — Supplementary Material 1 [file 40644_2025_831_MOESM1_ESM.docx]

## Table S1. MRI Scanning Protocol and Acquisition Parameters for Transverse T2WI

|  | Our Center | The Externa Set 1 | The Externa Set 2 | The Externa Set 3 | The Externa Set 4 | The Externa Set 5 |
| --- | --- | --- | --- | --- | --- | --- |
| Patients (n) | 111 | 4 | 8 | 8 | 16 | 13 |
| Manufacturer | UIH, uMR 770 | SIEMENS, Avanto | SIEMENS, Verio | SIEMENS, Aera | SIEMENS, Verio | GE MEDICAL SYSTEMS, SIGNA Architect |
| MFS | 3T | 1.5T | 3T | 1.5T | 3T | 3T |
| Sequence | T2WI | T2WI | T2WI | T2WI | T2WI | T2WI |
| TR (ms) | 3400 | 1000 | 3580 | 4820 | 5000 | 6000 |
| TE (ms) | 100 | 90 | 87 | 88 | 97 | 170 |
| ETL | 18 | 256 | 25 | 18 | 28 | 30 |
| Pixel Bandwidth (Hz/pixel) | 220 | 425 | 200 | 200 | 260 | 245 |
| Acquisition Matrix | 288\0\0\288 | 256\0\0\205 | 256\0\0\230 | 0\384\288\0 | 320\0\0\320 | 0\352\192\0 |
| Slice Thickness (mm) | 4.0 mm | 6.0 mm | 4.0mm | 6.0 mm | 5.0 mm | 4.0 mm |

Magnetic field strength; TR: repetition time; TE: echo time; ETL: echo train length

Our Center: The First Affiliated Hospital of Nanjing Medical University; The Externa Set 1: Union Hospital, Tongji Medical College, Huazhong University of Science and Technology; The Externa Set 2: Affiliated Hospital of Nanjing University of Traditional Chinese Medicine; The Externa Set 3: The Affiliated Suzhou Hospital of Nanjing Medical University; The Externa Set 4: The Affiliated Huai'an No. 1 People's Hospital of Nanjing Medical University; The Externa Set 5: Yixing People's Hospital.
